# Polymorph-Induced Reducibility and Electron Trapping Energetics of Nb and W Dopants in TiO_2_

**DOI:** 10.1021/acs.jpcc.5c04364

**Published:** 2025-08-19

**Authors:** Amit Chaudhari, Andrew J. Logsdail, Andrea Folli

**Affiliations:** † Cardiff Catalysis Institute, School of Chemistry, Translational Research Hub, 2112 Cardiff University, Maindy Road, Cardiff CF24 4HF, U.K.; ‡ Net Zero Innovation Institute, Cardiff Catalysis Institute, School of Chemistry, Translational Research Hub, 2112 Cardiff University, Maindy Road, Cardiff CF24 4HF, U.K.

## Abstract

Controlling the formation of electron polarons in TiO_2_ doped with transition metals is important for the design of transparent conducting oxides for high-efficiency photovoltaics and photocatalysts with tunable reaction selectivities. In this work, EPR spectroscopy is combined with Hubbard-corrected density functional theory (DFT+*U*), with refined atomic-like Hubbard projectors, to show the sensitivity of charge compensation in substitutionally doped Nb-TiO_2_ and W-TiO_2_ with respect to the TiO_2_ polymorph (*i.e.*, anatase or rutile). Both EPR magnetic tensors and DFT+*U-*predicted Nb 4*d* and W 5*d* orbital occupancies show the formation of differing dopant charge states depending on the TiO_2_ polymorph, with nonmagnetic Nb^5+^ and W^6+^ in doped anatase and paramagnetic Nb^4+^ and W^5+^ in doped rutile. The results provide an example of how a coherent experimental and theory-validated framework can be used to understand and predict the reducibility of dopants and electron trapping energetics in TiO_2_ polymorphs. The outcome enables greater control over the electronic and magnetic properties of metal oxide semiconductors, which are crucial for the rational design of next-generation materials for energy conversion and catalytic applications.

## Graphical abstract

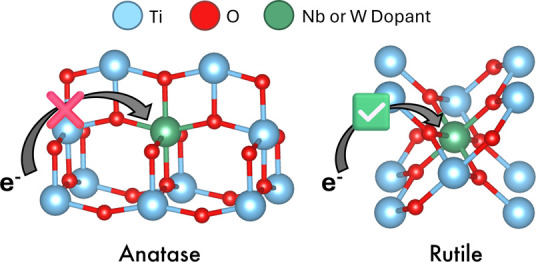

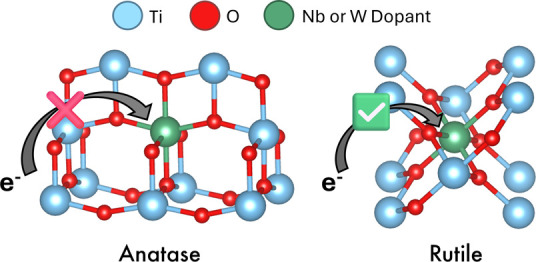

## 1 Introduction

Transparent conducting oxides (TCOs) underpin modern consumer electronics, photovoltaics, and light-emitting devices (LED and OLED). For example, anatase Nb-doped TiO_2_ (NTO) has emerged^1–10^ as a more sustainable alternative to the widely used indium tin oxide,^11–14^ capable of a resistivity of 2 × 10^–4^ to 3 × 10^–4^ Ωcm and 97% internal transmittance under visible light at room temperature for a 40 nm-thick film of anatase NTO with 3%_at_ Nb.^1^ In contrast, rutile NTO is more resistive,^2^ which allows for tailored applications based on the choice of TiO_2_ polymorph. Similarly, anatase W-doped TiO_2_ (WTO) exhibits an n-type metallic behavior^15,16^ with a resistivity of 1.5 × 10^–2^ Ωcm at room temperature for films with a doping concentration of 6.3%_at_.^15^ WTO has potential for TCO applications in electron transport layers in perovskite solar cells, with a demonstrated efficiency 28× greater than undoped TiO_2_.^17^ NTO and WTO have also attracted attention as promising photocatalysts with a 65% increase in photocurrent demonstrated for NTO nanorod photoelectrodes doped with 0.25%_at_ Nb when compared to undoped TiO_2_.^18^ NTO nanostructures, ranging from rutile nanorods to anatase nanosheets, also show improved adsorption of molecular O_2_ and the formation of superoxide radicals O_2_^–·^ under irradiation when compared to pristine TiO_2_ with the same morphology.^19^ Furthermore, both NTO and WTO nanostructures show enhanced dye photodegradation,^20,21^ photooxidation of nitrogen oxides (NO_*x*_) to nitrates,^22^ and ozone gas sensing^20^ when compared to undoped TiO_2_.

To understand and further optimize the performance of NTO and WTO, it is necessary to characterize and model the behavior of electrons and holes that contribute to material conductivity and chemistry. This includes the mobility of charge carriers within the bands (conduction and valence, respectively), their recombination, their trapping (forming electron polarons), and the charge carrier transfer mechanisms that drive redox processes when these materials are used as semiconductor photocatalysts. Given the electronic spin associated with electrons and holes, electron paramagnetic resonance (EPR) spectroscopy is a powerful tool for the precise identification and characterization of the dynamics, lifetimes, and spatial distribution of excitons and their trapping states within NTO and WTO, including the paramagnetic species following insertion of Nb and W dopants in the TiO_2_ lattice. For example, electron trapping states are mostly associated with Nb^4+^ and W^5+^ species, which are both paramagnetic *d* ^1^ species that can be detected and interrogated by EPR spectroscopy. The formation of Nb^4+^ and W^5+^ versus *vs.* Nb^5+^ and W^6+^ is particularly important as it directly influences conductivity and photocatalytic efficiency.

The literature is conflicted with respect to the lack of Nb^4+^ and W^5+^ EPR signals when substitutional Nb and W are introduced in anatase TiO_2_, in contrast to the presence of Nb^4+^ and W^5+^ EPR signals in doped rutile TiO_2_. There is currently no clear explanation of this observation, and so far, first-principles atomistic modeling methods like density functional theory (DFT) have not completely clarified these experimental observations. For example, geometry optimization with semilocal DFT followed by a single point calculation using a screen exchange hybrid functional (sX) predicts shallow donor states that are largely delocalized over Ti sites in anatase NTO,^5^ supporting resonant photoemission experiments, which confirm the absence of midgap states for anatase NTO.^2,23^ However, the same DFT calculations also predict a deep localized state in rutile NTO that is 0.9 eV below the conduction band edge, involving Ti 3*d* *_xy_* orbitals with a small contribution from Nb 4*d* orbitals, thus contradicting the EPR observations.^5^ Hubbard-corrected density functional theory (DFT+*U*) calculations predict Ti 3*d* midgap states in rutile NTO,^24^ but reports vary, with some predicting shallow donor states in rutile NTO^25^ and deep Ti 3*d* states in both anatase NTO^25,26^ and anatase WTO,^26^ *i.e.*, contradicting results. These computational results, based on DFT+*U* using planewave basis sets, fail to provide an unambiguous interpretation/prediction of the experimental observations; however, hybrid-DFT has been demonstrated to successfully predict W 5*d* midgap states in rutile WTO when using all-electron atom-centered basis functions to describe the wave function near the nucleus (in the full-potential linear augmented plane wave approach), and this gives promise for application of alternative basis representations.^27^

In this work, experimental EPR spectra for powder anatase and rutile NTO and WTO are presented, including the magnetic tensors characterizing Nb^4+^ and W^5+^ polarons in doped rutile, alongside DFT+*U* simulations in an all-electron numerical atom-centered orbital (NAO) framework^28^ to successfully simulate the experimental EPR observations. The DFT+*U* simulations use a refined atomic-like Ti 3*d* Hubbard projector, as well as the “*occupation matrix control*” (OMC) method,^29^ for controlling the Ti 3*d*, Nb 4*d,* and W 5*d* orbital occupancies through the self-consistent optimization of the system electronic structure towards the ground state. The presented combination of experiment and theory improves our fundamental understanding of the nature and formation of reduced species in semiconductor metal oxides, enabling the rational design of superior TCOs and heterogeneous photocatalysts.

## 2 Methodology

### 2.1 Material Synthesis

The NTO and WTO materials studied in this work were synthesized *via* a sol–gel route. 10 mL of titanium isopropoxide (≥97%, Sigma-Aldrich) was dissolved in 10 mL of anhydrous ethanol. After thorough mixing, 5 mL of deionized water (18 MΩcm) was slowly added to the solution. The resulting white precipitate redissolved upon further stirring. In the next step, 20 mL of a pH 10 ammonia/ammonium chloride buffer (5% ammonia, Sigma-Aldrich) was added to the solution. Finally, the desired amount of ammonium tungstate (BDH Chemicals) or ammonium niobate (V) oxalate hydrate (BDH Chemicals) to achieve a nominal 0.1 or 1.0 atom % was dissolved in 10 mL of warm deionized water and subsequently added to the solution. After thorough stirring for at least 4 h, the solution was filtered, washed several times with deionized water, and then dried at 60 °C for 4 h. The dry powders were ground in an agate mortar and transferred into a crucible for calcination. The samples were calcined at 600 °C for 4 h and then ground again afterward.

### 2.2 Powder X-ray Diffraction

To confirm mineralogy and crystallinity, X-ray diffraction (XRD) patterns were obtained using a Bruker D8 Advance diffractometer equipped to deliver CuKα_1_ X-ray radiation (1.54 Å) at room temperature. Refinement of the powder XRD patterns was carried out using the Profex suite for XRD.^30^

### 2.3 EPR Spectroscopy

X-band continuous-wave (CW) EPR spectra were recorded on a Bruker Elexsys E500 spectrometer equipped with an Oxford Instruments liquid-helium cryostat and a Bruker ER4122 SHQE-W1 superhigh Q resonator operating at 50 K. Before each measurement, the samples were evacuated for at least 12 h at 393 K and under a dynamic vacuum at ca. 1 × 10^–4^ bar. Experimental spectra were simulated using the EasySpin toolbox^31^ for Mathworks Matlab.

### 2.4 Electronic Structure Calculations

#### 2.4.1 DFT

All electronic structure calculations were performed using the Fritz-Haber Institute *ab initio* materials simulation (FHI-aims) software package,^32^ which uses an all-electron NAO basis set, interfaced with the Python-based atomic simulation environment (ASE).^33^ The standard light basis set (2020) was used, with equivalent accuracy to the TZVP Gaussian-type orbital basis set,^34^ as decided after benchmarking the TiO_2_ formation energy (see the Supplementary Information, SI, Section S1.1). Relativistic effects were accounted for using the zeroth-order regular approximation^32^ as a scalar correction, while the system charge and spin were set to zero. Periodic boundary conditions were applied using converged **k**-point sampling for the optimized anatase and rutile unit cell, separately (see SI Section S1.1). The mBEEF meta-GGA exchange correlation functional was used,^35,36^ as defined in Libxc,^37^ providing the best balance of accuracy and cost compared to other local, semilocal, and hybrid functionals (see SI Section S1.1). Self-consistent field (SCF) optimization of the electronic structure was achieved using a convergence criterion of 1 × 10^–6^ eV for the change in total energy, 1 × 10^–4^ eV for the change in the sum of eigenvalues, and 1 × 10^–6^ e a_0_^–3^ for the change in charge density. Geometry optimization used the quasi-Newton BFGS algorithm^38–41^ with a force convergence criterion of 0.01 eV/Å. Point defect calculations were performed using a 3 × 3 × 3 TiO_2_ supercell containing 324 and 162 atoms for anatase and rutile, respectively. The supercell size avoids spurious long-range defect–defect interactions between periodic images while simulating at low defect concentrations of 0.308% (anatase) and 0.617% (rutile) following substitution of a Ti atom with a Nb or W atom. Defect energies were calculated as

$$\Delta E_{\mathrm{Defect}}=E_{\mathrm{Defective}\mathrm{Bulk}{\mathrm{TiO}}_{2}}+\mu_{\mathrm{Ti}}-E_{\mathrm{Stoichiometric}\mathrm{Bulk}{\mathrm{TiO}}_{2}}-\mu_{\mathrm{Dopant}}$$ 1

For these systems, the chemical potential μ was calculated using the energy of bulk Ti (hexagonal close-packed structure), Nb (body-centered cubic structure), and W (body-centered cubic structure). All defect energies are listed in SI Section S1.3.3.

#### 2.4.2 DFT+*U*

All DFT+*U* calculations were performed using the on-site definition of the occupation matrix and the fully localized limit double counting correction.^28^ A Hubbard correction was applied to correct for the Coulomb self-interaction of Ti 3*d* orbital electrons only. Both the Hubbard *U* value and the occupation matrix (**n**_I_^σ^), which contains orbital occupancies for every spin channel (σ) for every atom (I), are used to calculate the corrective Hubbard term (*E*_*U*_^0^) as^28^

$$E_{U}^{0}[n_{I}^{\sigma }]=\sum_{\left(\sigma ,I\right)}U^{I}[\mathrm{Tr}(n_{I}^{\sigma })-\mathrm{Tr}(n_{I}^{\sigma }n_{I}^{\sigma })]$$ 2

Once *E*_*U*_^0^ is calculated, the total energy of the system (*E*_DFT+*U*_) is calculated using the DFT+*U* Hamiltonian, where *ρ*(**r**) is the electron density, *E*_DFT_ is the total energy of the system accounting for localized and delocalized orbitals, and *E*_*U*_^dc^ prevents the double counting of localized states in both *E*_DFT_ and *E*_*U*_^0^:^28^

$$E_{\mathrm{DFT}+U}\left[\rho \left(r\right)\right]=E_{\mathrm{DFT}}\left[\rho \left(r\right)\right]+E_{U}^{0}\left[n_{I}^{\sigma }\right]-E_{U}^{\mathrm{dc}}\left[n_{I}^{\sigma }\right]$$ 3

The occupation matrix is calculated by projecting all DFT-predicted Kohn–Sham states onto atomic-like orbitals defined by a Hubbard projector.^28^ However, using the default atomic Ti 3*d* Hubbard projector presented challenges in identifying the ground-state electronic structures of anatase and rutile NTO and WTO due to numerical instability. Therefore, constrained and self-consistent DFT+*U* calculations were performed with an atomic and modified Ti 3*d* Hubbard projector, respectively. Constrained DFT+*U* calculations were performed using the default atomic Ti 3*d* Hubbard projector and a Ti 3*d* Hubbard *U* value of 3 eV in anatase TiO_2_ and 4 eV in rutile TiO_2_. These Hubbard *U* values were chosen to minimize the average error in the DFT+*U*-predicted band gap and unit cell equilibrium volume, calculated by fitting to the Birch–Murnaghan equation of state using ASE,^42^ versus experimental references (see SI Section S1.2).^43,44^ Here, the OMC method^29^ was used to *fix* the polaron(s) at specific atom(s) by modifying the corresponding atomic orbital occupation matrix (see SI Section S1.3.1).

Next, self-consistent DFT+*U* calculations were performed with a refined atomic-like Ti 3*d* Hubbard projector. Here, the “*occupation matrix release*” method^28^ was used to *initialize* the polaron(s) at specific atom(s) before a two-step optimization of the system electronic structure until full geometry optimization was achieved (see SI Section S1.3.1). A refined atomic-like Ti 3*d* Hubbard projector was defined as a linear combination of the atomic Ti 3*d* and hydrogenic auxiliary basis function in the light basis set, where the auxiliary function is subject to a Gram–Schmidt orthogonalization with respect to the atomic function, with the corresponding linear combination expansion coefficients *c*_1_ = 0.828 and *c*_2_ = −0.561. The values of *c*_1_ and *c*_2_ were chosen based on the work of Jakob and Oberhofer, who computed a Ti 3*d* Hubbard projector for bulk rutile TiO_2_ in FHI-aims from first-principles.^45^ These coefficients enabled successful convergence to the ground state using a Ti 3*d* Hubbard *U* value of 3 eV for both anatase and rutile NTO and WTO. In general, tuning the Hubbard projector in FHI-aims to navigate numerical instability in the simulation of defective transition metal oxides has not yet been systematically investigated and will be the focus of a future study.

## 3 Results and Discussion

### 3.1 Polymorphism and Dopant Loadings

The refined X-ray powder diffraction patterns, presented in SI Section S2, show that 0.1%_at._ Nb or W in TiO_2_, synthesized *via* a sol–gel route and calcined at 600 K, allows for the formation of mixed anatase and rutile polymorphs. The same result occurs for undoped TiO_2_, which exhibits a rutilization temperature higher than 500 K (calcination of sol–gel TiO_2_ precursors with 0.1%_at._ Nb or W at *T* < 500 K generates anatase-only polymorphs^9,46,47^ ). Refinement of the XRD patterns (SI Section S2) reveals 92% anatase and 8% rutile for NTO and 72% anatase and 28% rutile for WTO. These samples are referred to as NTO-AR and WTO-AR herein. On the contrary, 1.0%_at._ of the same dopants in conjunction with calcination at 600 K allows for the formation of anatase-only NTO and WTO (SI Section S2). These samples are referred to as NTO-A and WTO-A herein.

### 3.2 Experimentally Detected Electron Trapping States in Anatase and Rutile NTO and WTO

Electron and hole trapping are normally single electron transfer events occurring within the material. As such, they can be followed experimentally by detecting the formation or disappearance of paramagnetic species using EPR spectroscopy. Nb^5+^ ([Kr]­4*d* ^0^) and W^6+^ ([Xe]­5*d* ^0^) replace Ti^4+^ ([Ar]­3*d* ^0^) in the TiO_2_ lattice aliovalently and isomorphically, as Nb^5+^, W^6+^, and Ti^4+^ have almost identical octahedral coordination environments with ionic radii of 78 pm, 74 pm, and 74.5 pm, respectively. The dopant incorporation reactions for extrinsic defects using the Kröger–Vink notation can be written as follows:

$${\mathrm{Nb}}_{2}O_{5}\overset{2{\mathrm{TiO}}_{2}}{\to }2{\mathrm{Nb}}_{\mathrm{Ti}}^{.}+4O_{O}^{x}+2e^{\prime }+\frac{1}{2}O_{2}$$ 4

$$2{\mathrm{Nb}}_{2}O_{5}\overset{5{\mathrm{TiO}}_{2}}{\to }4{\mathrm{Nb}}_{\mathrm{Ti}}^{.}+V_{\mathrm{Ti}}^{⁗}+10O_{O}^{x}$$ 5

$${\mathrm{WO}}_{3}\overset{{\mathrm{TiO}}_{2}}{\to }W_{\mathrm{Ti}}^{..}+2O_{O}^{x}+2e^{\prime }+\frac{1}{2}O_{2}$$ 6

$$2{\mathrm{WO}}_{3}\overset{3{\mathrm{TiO}}_{2}}{\to }2W_{\mathrm{Ti}}^{..}+V_{\mathrm{Ti}}^{⁗}+6O_{O}^{x}$$ 7

Valence-induced electron formation, as highlighted by eqs 4 and 6, increases the n-type character of NTO and WTO compared to that of undoped TiO_2_. Our EPR attempts at identifying *substitutional* and *isolated* Nb^4+^ and W^5+^ in NTO-A and WTO-A failed, which corroborates with previous experiments by De Trizio et al., which could not detect Nb^4+^ in Nb-doped colloidal anatase nanocrystals even at liquid-helium temperature.^6^ Giamello and co-workers^8^ and ourselves^9^ independently showed that, in the case of Nb doping in anatase-only TiO_2_, Ti^3+^ is mostly formed as a result of valence induction when Nb^5+^ aliovalently replaces Ti^4+^ in the anatase lattice:

$${\mathrm{Ti}}_{\mathrm{Ti}}^{x}+e^{\prime }\to {\mathrm{Ti}}_{\mathrm{Ti}}^{\prime }$$ 8

The resulting Ti^3+^ exhibits an anisotropic EPR spectrum characterized by a ***g*** tensor with axial symmetry and principal values equal to *g*_⊥_ = 1.988 and *g*_//_ = 1.957,^8,9^ consistent with a highly delocalized bulk species that is responsible for causing an increased conductivity of the doped anatase TiO_2_.^8^ This Ti^3+^ is structurally and magnetically different from a surface-localized Ti^3+^ that forms following chemical or chemo/thermal reduction of undoped TiO_2_.^8^ The amount of delocalized bulk Ti^3+^ can also be augmented by photoinjection of extra conduction electrons^8,9^ followed by trapping:

$$\gamma \overset{{\mathrm{TiO}}_{2}}{\to }h_{\mathrm{vb}}^{+}+e_{\mathrm{cb}}^{-}$$ 9

The situation appears completely different in the cases of NTO-AR and WTO-AR, as demonstrated by the respective X-band CW EPR spectra reported in Figure 1a (NTO-AR) and Figure 1b (WTO-AR).

**Figure 1:**
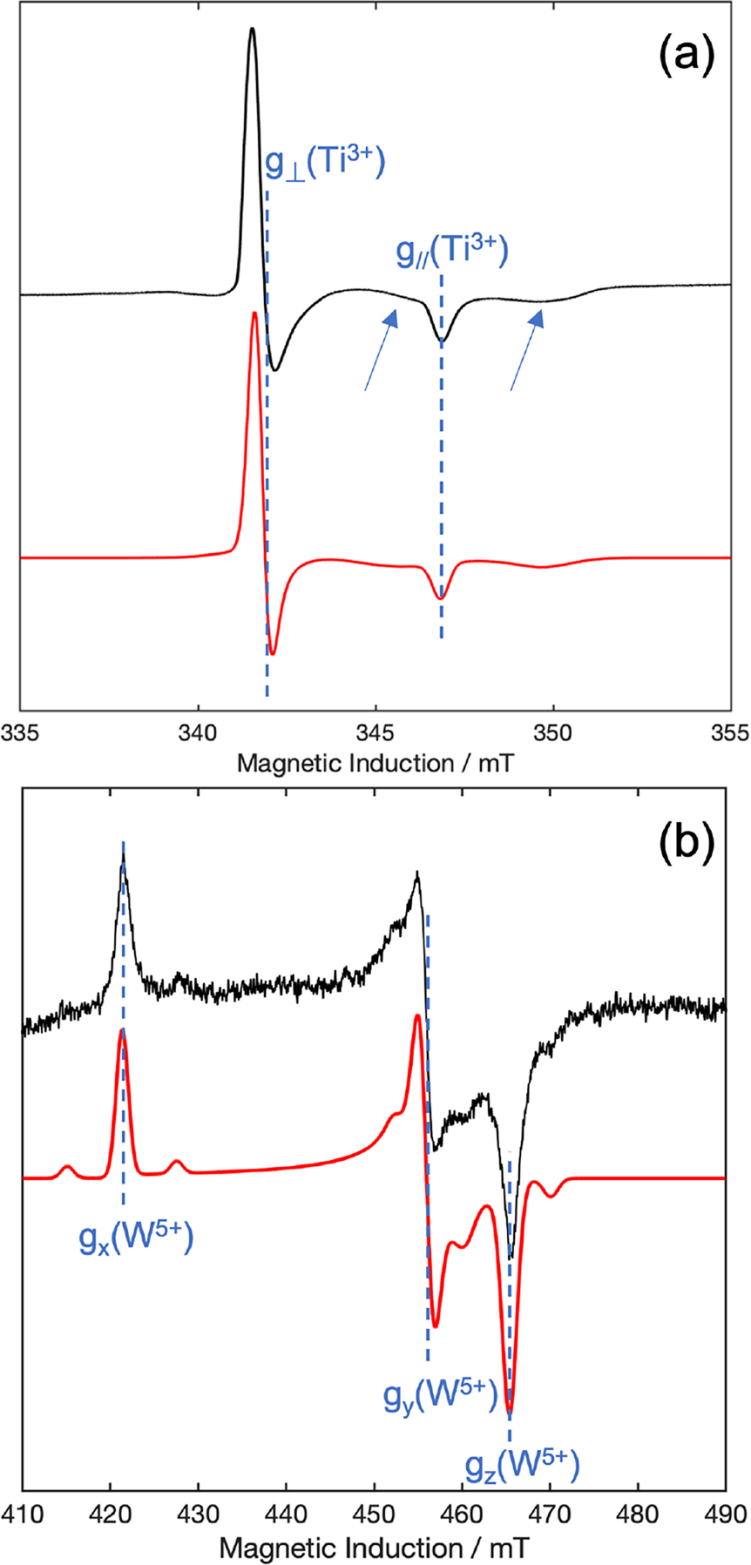
X-band CW EPR spectra at 50 K for (a) Nb-doped and (b) W-doped mixed-polymorph TiO_2_ nanoparticles with a doping concentration of 0.1%_at._ (NTO-AR and WTO-AR, respectively).

The clear axial signal in the spectrum of NTO-AR in Figure 1a can be attributed to bulk Ti^3+^, as previously described for the case of solely the anatase polymorph. A very broad and low-intensity signal is also visible at 50 K as shown by the arrows in Figure 1a. We propose that this broad signal is associated with Nb^4+^ in rutile. This assignment can be explained as follows. Figure 2b,c show the simulated angular dependency of the single-crystal EPR spectra at 4.2 K of Nb^4+^ centers in pure rutile NTO at the X- and Q-bands, respectively, computed using experimental values derived from a rutile single crystal.^48^

**Figure 2:**
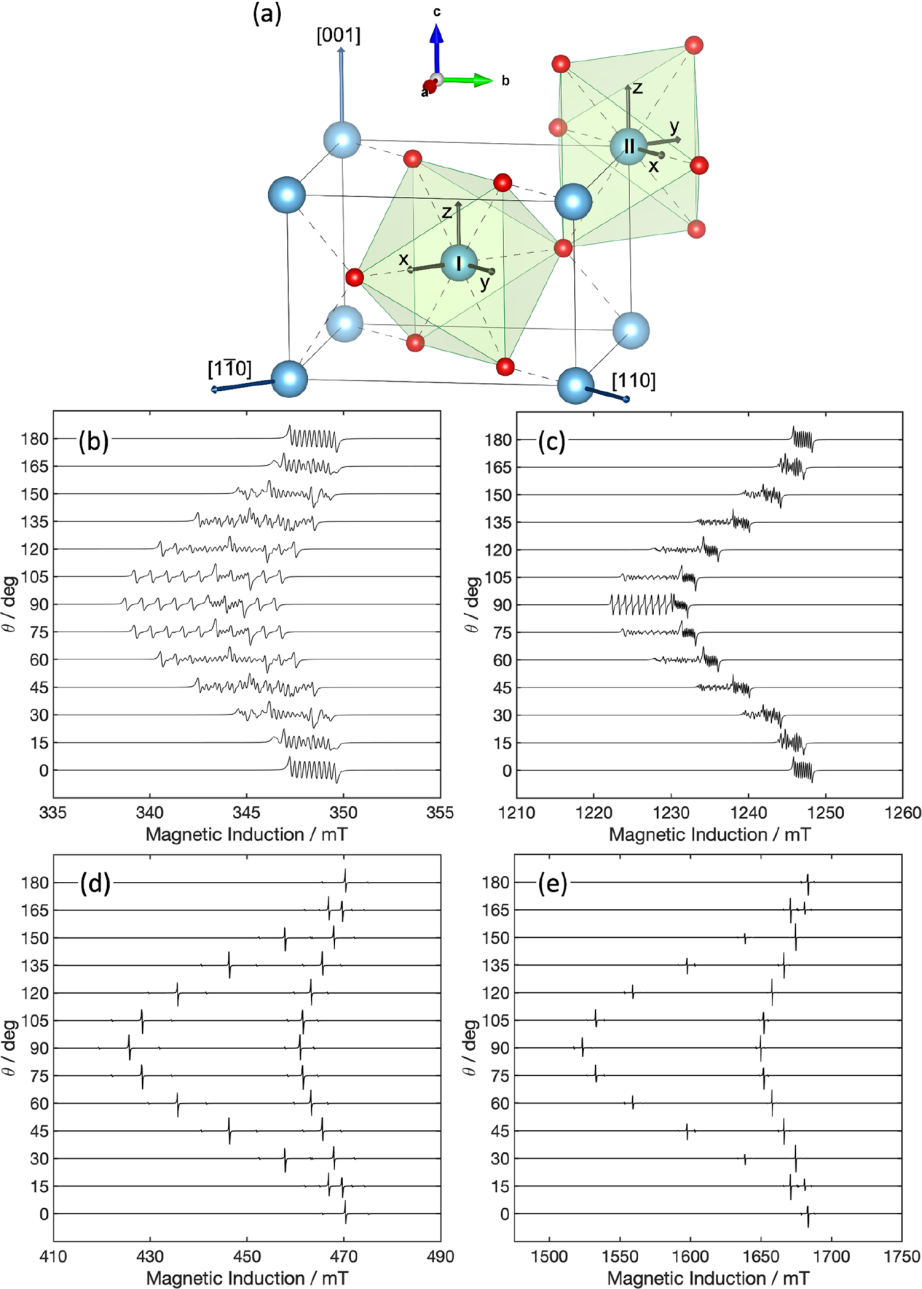
(a) Tetragonal crystal structure of rutile TiO_2_ with two inequivalent Ti atoms (I and II). (b)–(e) Simulated angular dependency of the rutile single-crystal EPR spectra at 4.2 K for Nb^4+^ centers at (b) X-band and (c) Q-band; and W^5+^ centers at (d) X-band and (e) Q-band.

The crystal structure of rutile TiO_2_ in Figure 2a is tetragonal (*D*_4*h*_^14^, space group P42/mnm) with two Ti per unit cell, which are equivalent except for a π/2 rotation about the *c* axis, as also evidenced by the orientation of the eigenframes of the magnetic tensors reported for the two inequivalent sites I and II in Figure 2a. The two Ti sites possess orthorhombic point symmetry *D*_2*h*_. Details of the spin Hamiltonian used for the simulations can be found in Table 1.

**Table 1:** Spin Hamiltonian Parameters of Reduced Dopant Metal Centers Detected in Nb^5+^ and W^6+^ Doped TiO_2_.^a^

Reduced metal center	TiO_2_ polymorph	*g*_*x*_	*g*_*y*_	*g*_*z*_	*A*_*x*_ (MHz)	*A* *_y_* (MHz)	*A* *_z_* (MHz)	Reference
Nb^4+^	rutile	1.970	1.985	1.941	n.d.	n.d.	n.d.	This work
Nb^4+^	rutile	1.973	1.981	1.948	5.0	23.8	7.0	^48^
W^5+^	rutile	1.594	1.473	1.443	277.5	122.4	191.1	This work
W^5+^	rutile	1.5944	1.4725	1.4431	277.3	122.3	191.0	^49^

a *x*, *y*, and *z* are here along [11̅0], [110], and [001], respectively. [001] corresponds to the rutile crystal *c*-axis (see also Figure 2).

The *g*_*x*_ and *g*_*y*_ completely overlap at X-band frequencies, but they can be resolved at Q-band frequencies. Hyperfine interaction of the 4*d* ^1^ unpaired electron with the ^93^Nb nucleus is expected (*I*(^93^Nb) = 9/2, 100% natural abundance), giving rise to 10 hyperfine lines. The *A*_*y*_ value is noticeably larger than *A*_*x*_ and *A*_*z*_, with some resolved structure due to δ*m*_I_ = ± 1 transitions that are allowed *via* the electric quadrupole interaction when **B** is not aligned to the principal axes of the crystal. The hyperfine structure has been shown to vanish above 25 K on the single crystal,^48^ with particularly the **B**//[001] and **B**//[11̅0] sets of lines coalescing into a single line already at 25 K.^48^ The increasingly dispersive character of the signal above 25 K and the line narrowing observed upon coalescing of the hyperfine structure were interpreted by Zimmermann^48^ as a combination of two contributions, *i.e.*, thermal excitation of 4*d* ^1^ donor electrons to the conduction band plus exchange scattering of 4*d* ^1^ donor electrons with the conduction band. Both of these contributions give rise to electron hopping from different donor sites (*i.e.*, Nb^4+^) via the conduction band (Anderson’s model of random frequency modulation). The activation energy for the hopping between the donor level and the conduction band was found to be *E*_a_/*k* = 72 K at temperatures below 40 K, while the same activation energy was much larger at temperatures up to 300 K, in agreement with more recent evidence suggesting that rutile NTO is resistive at room temperature.^2^ Moving from single crystal to powder samples, Kiwi et al.^50^ showed that, in a mixed anatase and rutile powder sample, a broad signal could be found at 4.2 K matching the ***g*** tensor reported by Zimmermann.^48^ Our broad signal in Figure 1a matches the signal reported by Kiwi et al.,^50^ although it is much broader due to the much higher temperature of our measurement, *i.e.*, 50 K (according to Zimmermann,^48^ the signal completely vanishes above 77 K).

$${\mathrm{Nb}}_{\mathrm{Ti}}^{.}+e^{\prime }\to {\mathrm{Nb}}_{\mathrm{Ti}}^{x}$$ 10

In the case of WTO-AR, the situation is very similar to that described above for NTO-AR. Figure 1b shows a clear anisotropic EPR spectrum characterized by a rhombic ***g*** tensor with principal values reported in Table 1. Small intensity doublets (*m*_*I*_ = ± 1/2 lines) are visible on each side of the three principal resonances due to hyperfine interaction of the 5*d* ^1^ unpaired electron with the ^183^W nucleus (*I*(^183^W) = 1/2, 14.3% natural abundance). The other naturally occurring isotopes of W are ^180^W, ^182^W, ^184^W, and ^186^W, all with nuclear spin quantum number *I* = 0, and these account for the three principal *m*_*I*_ = 0 resonance lines. W^5+^ in TiO_2_ is not affected by the same fast relaxation issues as Nb^4+^ and therefore well-defined EPR spectra can be easily obtained at 50 K, as visible in Figure 1b. The values of the magnetic tensors are in good agreement with those of Chang^49^ for W^5+^ centers in rutile single crystals. The angular dependency has been simulated in Figure 2d,e at the X- and Q-bands, respectively.

This combined evidence of NTO-AR and WTO-AR indicates that the reduction of Nb^5+^ and W^6+^ dopants in the TiO_2_ host lattice occurs only in the rutile polymorph.

### 3.3 Computed Electron Trapping States in Anatase and Rutile NTO and WTO

To rationalize the magnetic resonance observations in terms of the energetics of intraband gap states, we relied on self-consistent DFT+*U* calculations in an NAO framework, given that constrained DFT+*U* calculations could not rationalize our experimental observations (see SI Section S1.3.2). The total density of states (TDOS) reported in Figure 3a,c shows defect states pinned to the bottom of the TiO_2_ conduction band for both anatase NTO and WTO, respectively, in perfect agreement with our EPR observations for NTO-A and WTO-A. From the corresponding projected density of states (PDOS) in Figure 3e,g, there are small Nb 4*d* and W 5*d* signatures at the Fermi level indicating these states are partially delocalized over Ti sites, contributing to metallic-type behavior. On the other hand, self-consistent DFT+*U* calculations show that the Nb 4*d* signature in rutile NTO is an order of magnitude greater at the Fermi level than in anatase NTO, when normalizing with respect to the different defect concentrations in our simulation supercells, as shown in the PDOS in Figure 3e,f.

**Figure 3:**
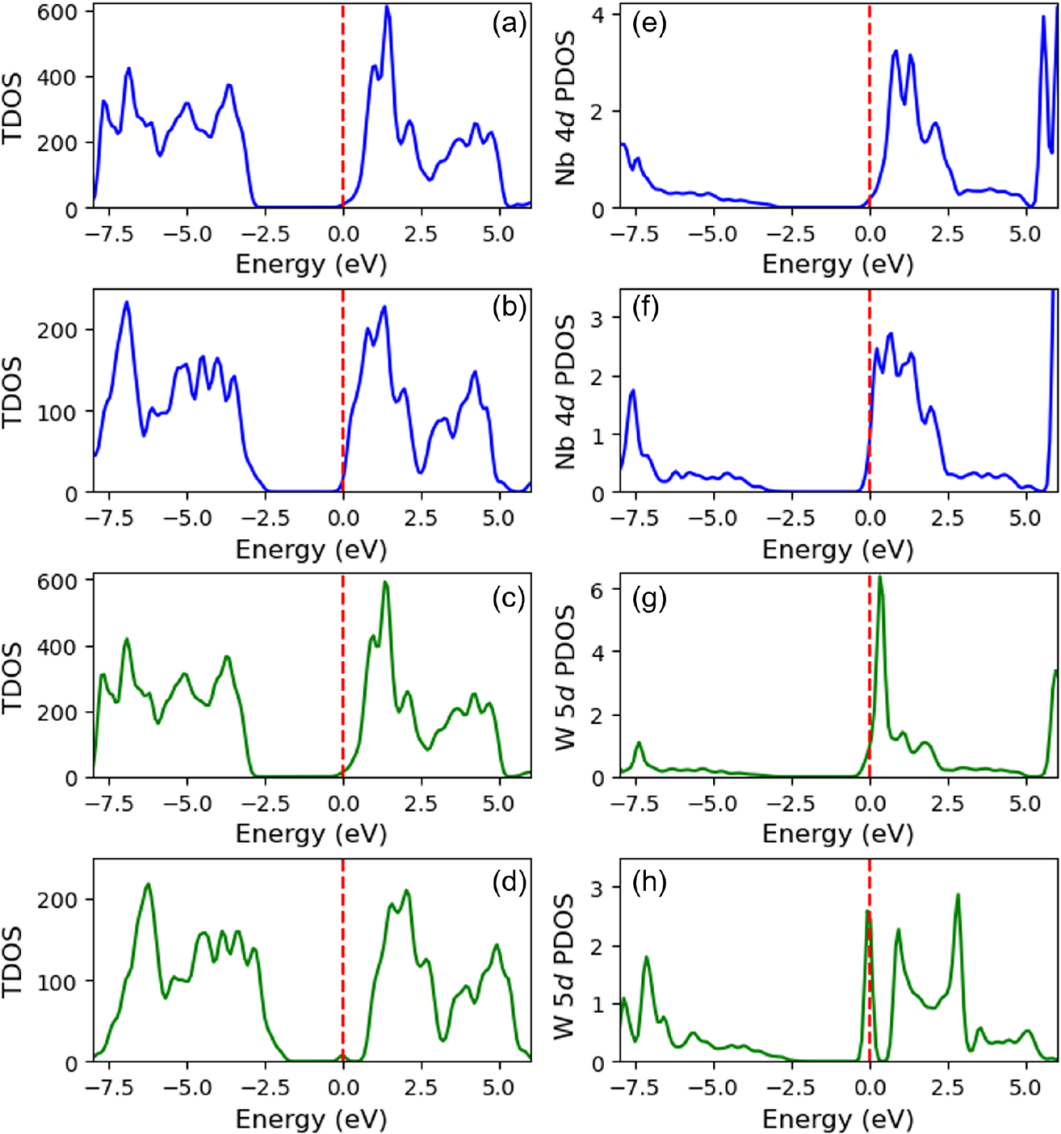
Self-consistent DFT+*U*-predicted TDOS and PDOS for anatase NTO ((a) and (e), respectively), anatase WTO ((b) and (f), respectively), rutile NTO ((c) and (g), respectively), and rutile WTO ((d) and (h), respectively). All TDOS and PDOS are plotted relative to the Fermi level indicated by the red dashed line.

These results can be attributed to differences in the filling of the five Nb 4*d* orbitals, notably, the greater occupancy of the three *t*_*2g*_ orbitals in rutile NTO, which correspond to orbital magnetic quantum numbers *m*_*l*_ = −2, −1, and 1 ^29^ in Figure 4. There is a negligible difference in the trace of the Nb 4*d* occupation matrix (*i.e.*, the total Nb 4*d* subshell occupancy) in anatase NTO (1.48) compared with that of rutile NTO (1.49). Figure 3d,h show the TDOS and W 5*d* PDOS for the rutile WTO, respectively. Here, a localized W 5*d* midgap state is predicted at ca. 0.7 eV below the TiO_2_ conduction band. The character of the midgap state is W 5*d*_*z* ^2^_, corresponding to a large occupation number of 0.93 for the *m*_l_ = 0 orbital in the W 5*d* occupation matrix (Figure 4). The other diagonal terms of the W 5*d* occupation matrix are of similar magnitude in anatase WTO and rutile WTO, which suggests the formation of W^5+^ in rutile but not in anatase.

**Figure 4:**
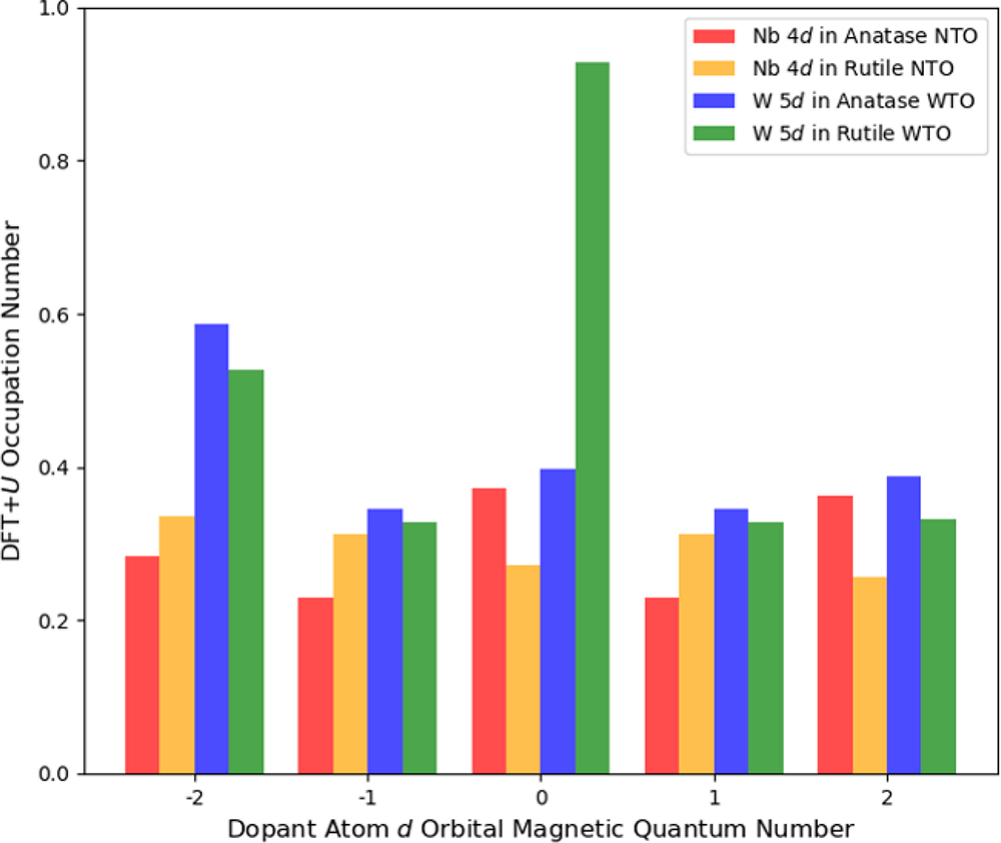
Ground-state orbital occupation numbers for Nb 4*d* and W 5*d* in doped anatase and rutile TiO_2_ calculated using self-consistent DFT+*U*.

The formation of W^5+^ in rutile WTO is also suggested based on the local lattice distortion surrounding the W dopant, which is associated with localized polaronic states in defective TiO_2_,^51,52^ as shown in Figure 5, which plots the change in the bond distance between the dopant atom and six neighboring Ti atoms relative to the average Ti–Ti bond distance in bulk anatase and rutile TiO_2_. Figure 5c shows symmetric geometric relaxation around the substitutional defect in anatase NTO and WTO, where the change in bond length between the dopant atom and Ti atoms A–D is almost constant for both materials, as is the change in bond length with Ti atoms E and F. In Figure 5d, there is a stronger asymmetric local lattice distortion around the dopant atom in rutile WTO compared to that in rutile NTO, as shown by the differences in the change in bond lengths between the dopant atom and Ti atoms G-L.

**Figure 5:**
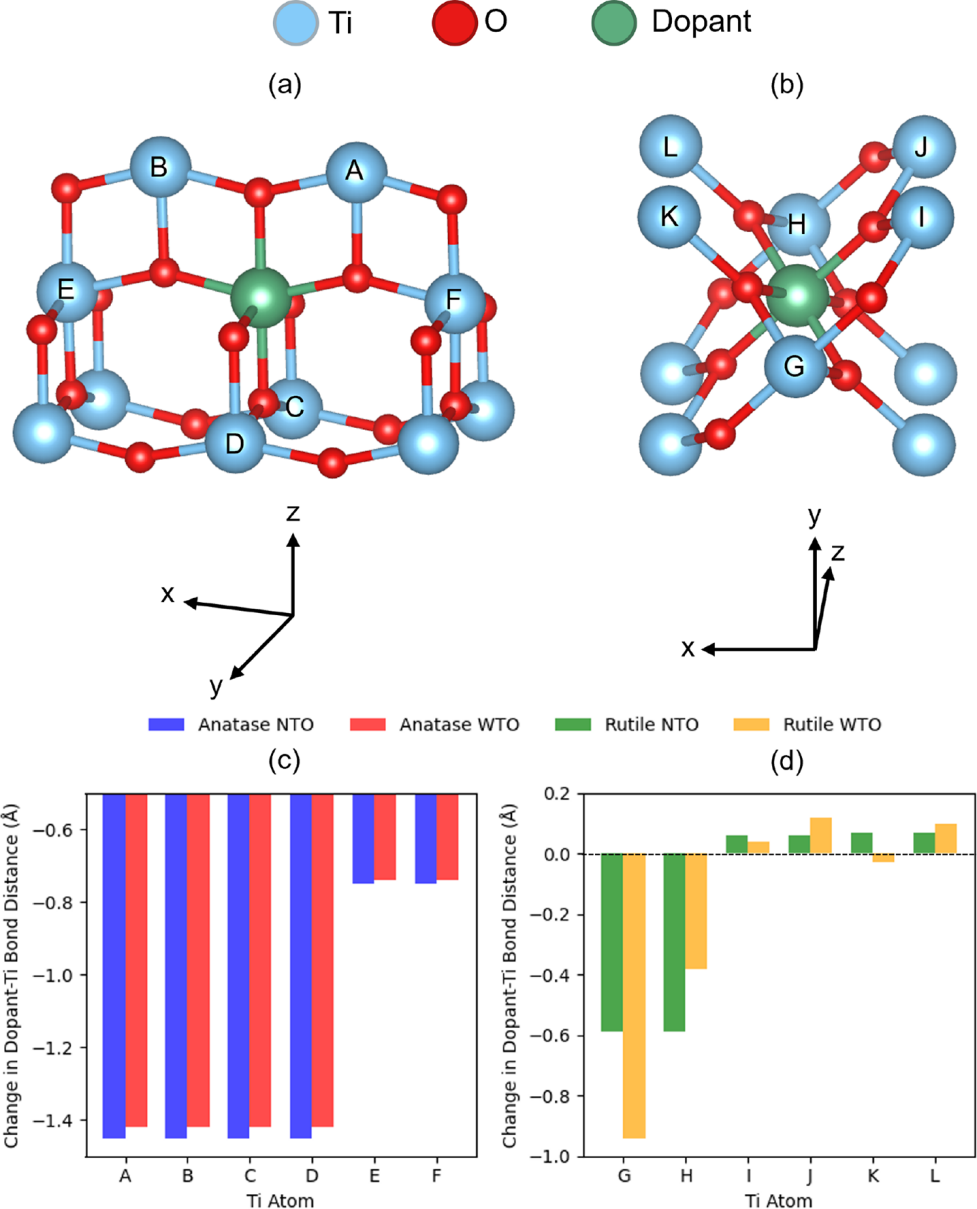
Change in the self-consistent DFT+*U-*calculated bond distances between the dopant atom and surrounding Ti atoms in doped anatase (atoms A–F in (a)) and doped rutile (atoms G–L in (b)) calculated relative to the average Ti–Ti bond distance in bulk anatase (c) and rutile (d) TiO_2_.

## 4 Conclusions

NTO and WTO are promising TCOs with applications in heterogeneous photocatalysis and high-efficiency photovoltaics and electronics. However, there remains uncertainty in the atomistic mechanisms that govern charge compensation in these materials, which prevents the development of accurate structure–property models for material optimization. Using EPR spectroscopy, charge compensation is shown as highly sensitive to the TiO_2_ polymorph, with Nb^4+^ and W^5+^ signals present in substitutionally doped rutile but not in doped anatase. The observations are complemented by contemporary DFT+*U* calculations in an all-electron NAO framework, for which self-consistent resolution of the Ti 3*d*, Nb 4*d,* and W 5*d* orbital occupancies is crucial. Self-consistent DFT+*U* predicts the favorability of Nb^4+^ in rutile NTO through greater filling of the Nb 4*d t*_*2g*_ orbitals and reduced filling of the *e*_*g*_ orbitals compared to anatase NTO. Self-consistent DFT+*U* also predicts W^5+^ in rutile WTO through the formation of a localized midgap state of 5*d*_*z* ^2^_ character that is not formed in anatase WTO. Our approach and findings provide a coherent view on the reducibility of metal centers in semiconducting TiO_2_, matching experiments and theory without apparent disagreement, while also providing a clear understanding of how the reducibility of metal centers and electron trapping energetics in TiO_2_ are polymorph-dependent. The improved fundamental understanding enables precise control over the electronic and magnetic properties of transition-metal-doped TiO_2_, advancing the rational design of high-performance materials for energy conversion and catalytic applications.

## Supplementary Material



## Data Availability

The input and output files of all electronic structure calculations have been uploaded as a data set to the NOMAD repository at doi: 10.17172/NOMAD/2024.09.04-1.

## Notes

Section S1, electronic structure calculations; and Section S2, powder XRD characterization (PDF)
