# Supplementary material for: Polymorph-Induced Reducibility and Electron Trapping Energetics of Nb and W Dopants in TiO2
Source: J Phys Chem C Nanomater Interfaces. 2025 Aug 19;129(34):15453–61. doi: 10.1021/acs.jpcc.5c04364 (PMC12400418; doi:10.1021/acs.jpcc.5c04364)
Supplement: Supplementary file 1 [file jp5c04364_si_001.pdf]

Polymorph-Induced Reducibility and Electron Trapping Energetics of Nb and W Dopants in TiO_2: Supplementary Information

Amit Chaudhari [a], Andrew J. Logsdail [a], Andrea Folli *[a,b]

[a] Cardiff Catalysis Institute, School of Chemistry, Cardiff University, Translational Research Hub, Maindy Road, Cardiff, CF24 4HF, United Kingdom.

[b] Net Zero Innovation Institute, Cardiff Catalysis Institute, School of Chemistry, Cardiff University, Translational Research Hub, Maindy Road, Cardiff, CF24 4HF, United Kingdom.

E-mail: FolliA@cardiff.ac.uk

S1 Electronic Structure Calculations

S1.1 DFT Benchmarking

TiO_2 formation energies were calculated using the energies of bulk Ti (in the hexagonal close packed, HCP, crystal structure) and an isolated O_2 molecule:

$$\Delta E_{\mathrm{Form}} = E_{\mathrm{TiO_2}} - E_{\mathrm{Ti}} - E_{\mathrm{O_2}} \tag{1}$$

The light basis set was chosen based on the negligible difference in the DFT-predicted ΔE_{Form}, as in Figure S1, whilst dramatically reducing the computational cost.

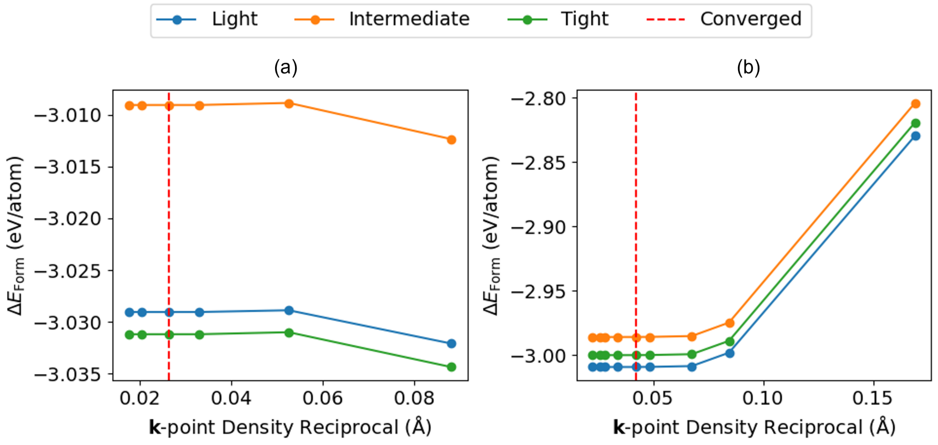

Figure S1: Variation of the bulk TiO_2 formation energy with respect to the reciprocal of the **k**-point density and basis set size (light, intermediate and tight), calculated using the PBE functional, for (a) anatase and (b) rutile. The red dashed line corresponds to the converged **k**-point density reciprocal.

The mBEEF meta-GGA exchange correlation functional provided the best accuracy in the DFT-predicted ΔE_{Form}, band gap (E_{bg}) and unit cell equilibrium volume (V_0), whilst providing a reasonable computational cost, as listed Table S1 and illustrated in Figure S2.

Table S1: Comparison of DFT exchange correlation functionals for predicting the formation energy, ΔE_{Form} (eV), band gap, E_{bg} (eV), unit cell equilibrium volume cell volume, V_0 (Å^3) for anatase and rutile TiO_2, as well as the CPU time per SCF cycle, t (s) for unit cell geometry optimisation.

Functional	ΔE_{Form}		E_{bg}		V_0		t	
	Rutile	Anatase	Rutile	Anatase	Rutile	Anatase	Rutile	Anatase
LDA	-3.42	-3.43	1.74	2.12	61.22	132.54	0.93	0.47
PBE	-3.01	-3.03	1.78	2.20	64.60	139.72	1.16	0.63
PBEsol	-3.12	-3.13	1.76	2.16	62.71	135.75	1.20	0.60
BLYP	-3.14	-3.17	1.76	2.21	66.26	143.21	1.23	0.63
M06-L	-3.12	-3.09	2.10	2.58	64.12	138.77	1.43	0.81
SCAN	-3.43	-3.42	2.08	2.59	63.08	136.41	1.48	0.78
rSCAN	-3.37	-3.37	2.11	2.59	62.95	136.18	1.51	0.79
mBEEF	-3.26	-3.28	2.21	2.65	63.50	137.26	1.69	0.94
B3LYP	-3.34	-3.36	3.56	3.93	63.98	138.30	108.05	32.69
PBE0	-3.20	-3.21	4.05	4.29	62.39	135.49	38.47	62.41

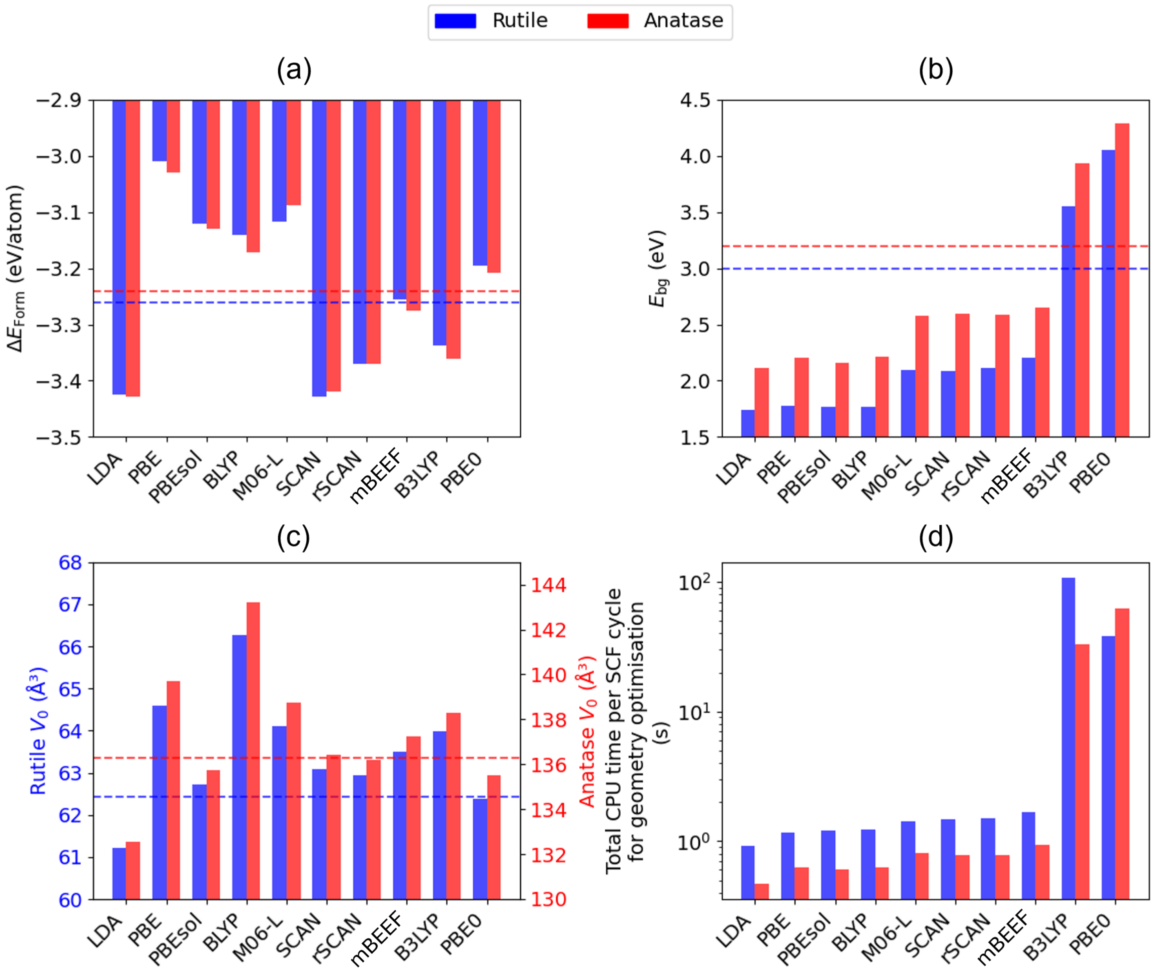

Figure S2: Comparing the DFT-predicted (a) formation energy, (b) band gap, (c) unit cell equilibrium volume and (d) CPU time per SCF cycle for unit cell geometry optimisation for bulk anatase and rutile TiO_2 using 10 different exchange correlation functionals. Experimental reference values are indicated by horizontal dashed lines[1–3]

S1.2 DFT+U Parameterisation

Constrained DFT+U calculations were performed using Hubbard U values of 3 eV and 4 eV to correct for the Coulomb self-interaction of Ti 3d orbital electrons in anatase and rutile TiO_2 respectively (using the default atomic Ti 3d Hubbard projector function). These values were chosen to minimise the average error (given by the Euclidean norm in Equation 2) in the DFT+U-predicted TiO_2 band gap and unit cell equilibrium volume, relative to experimental references[1,4] as illustrated in Figure S3.

$$\text{Average Error} = \left\| \frac{100 \times (E_{\text{bg}}^{\text{DFT}+U} - E_{\text{bg}}^{\text{Exp}})}{E_{\text{bg}}^{\text{Exp}}}, \frac{100 \times (V_0^{\text{DFT}+U} - V_0^{\text{Exp}})}{V_0^{\text{Exp}}} \right\| \tag{2}$$

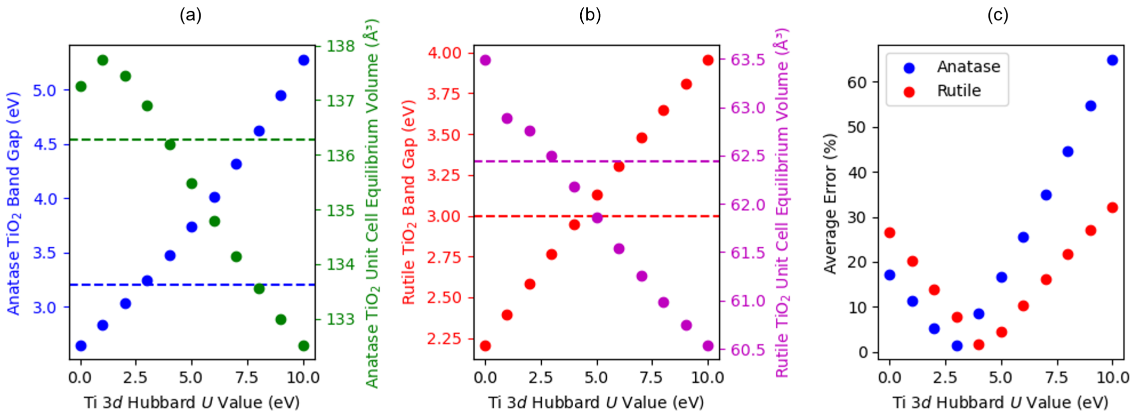

Figure S3: Benchmarking the Ti 3*d* Hubbard *U* value (using the default atomic Ti 3*d* Hubbard projector function) by comparing the DFT+*U*-predicted band gap and unit cell equilibrium volume of bulk (a) anatase and (b) rutile TiO_2 with experimental references[1,4] (dashed lines). (c) shows the average error in (a) and (b) at each *U* value.

S1.3 Defect Calculations

S1.3.1 Optimising Orbital Occupancies

The diagonal terms of the occupation matrix correspond to the orbital occupancy for a given magnetic quantum number (d_{m_l}); with d_{-2}, d_{-1}, d_0, d_1 and d_2 corresponding to the d_{xy}, d_{yz} d_{z^2} d_{xz} and $d_{x^2-y^2}$ orbitals, respectively.[5] In the constrained DFT+*U* calculations, the "*occupation matrix control*" method (OMC)[5] was used to initialise polaron(s) in the d_{z^2} orbital at nearest neighbour (or next nearest neighbour) Ti and/or dopant atom sites by setting d_0 to 1. In these calculations, the inclusion of a Hubbard correction for Nb 4*d* or W 5*d* orbital electrons was found to result in geometric instability due to forced overlocalisation of polarons in the system; therefore a Hubbard correction was not applied to these orbitals. In the self-consistent DFT+*U* calculations, the "*occupation matrix release*" (OMR) method[6] was used to ensure a numerically stable convergence to the ground state. This was achieved by initialising polaron(s) in the same way as the constrained DFT+*U* calculations, before the total energy (E) is pre-converged using OMC until $\Delta E \leq 0.001$ eV and then the OMC constraint is relaxed and the orbital occupancies are calculated self-consistently.[6]

S1.3.2 Constrained DFT+*U* Density of States

Constrained DFT+*U* simulations with the default atomic atomic Ti 3*d* Hubbard projector were not able to rationalise our EPR observations. Figures S4(a) and S4(b) show the total and projected density of states for rutile NTO, respectively, with occupation matrices initialised to reflect Nb^{4+}(Nb $4d^1$). Here, there is a localised defect state of Ti 3*d* character. Figures S4(c) and (d) show the total and projected density of states for rutile WTO, respectively, with occupation matrices initialised to reflect W^{5+}(W $5d^1$). Here, the Fermi level is pinned to the bottom of the conduction band, indicating the delocalisation of both Ti 3*d* and W 5*d* states.

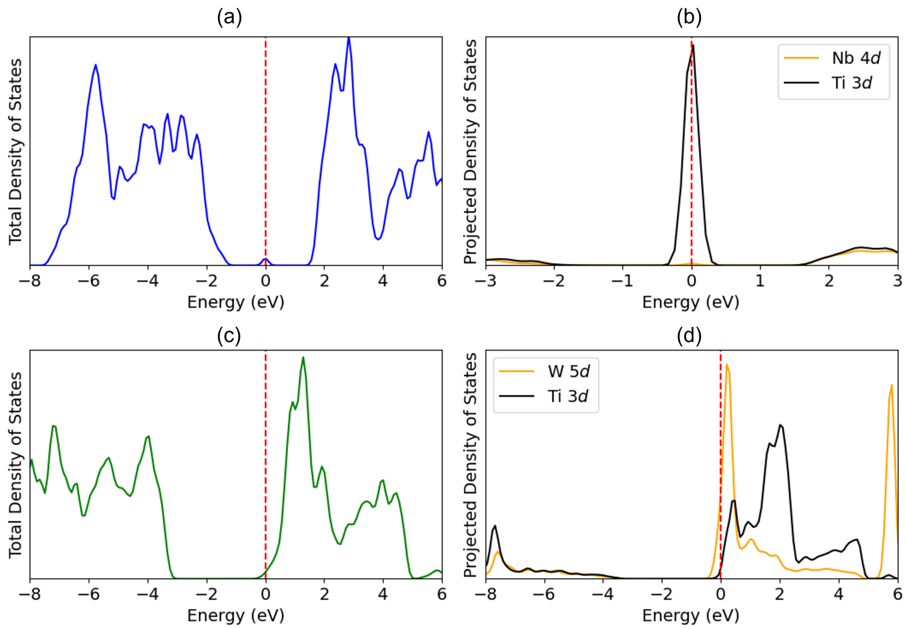

Figure S4: Total and projected density of states for rutile NTO ((a) and (b) respectively) and rutile WTO ((c) and (d) respectively) calculated using constrained DFT+U with the default atomic Ti 3d Hubbard projector (U = 3 eV for anatase and 4 eV for rutile, $c_1 = 1$ and $c_2 = 0$).

S1.3.3 Defect Energies

The substitutional defect energies for anatase and rutile NTO and WTO, are presented in Figures S5, calculated using DFT, constrained DFT+U using the default atomic Ti 3d Hubbard projector and self-consistent DFT+U using a refined atomic-like Ti 3d Hubbard projector.

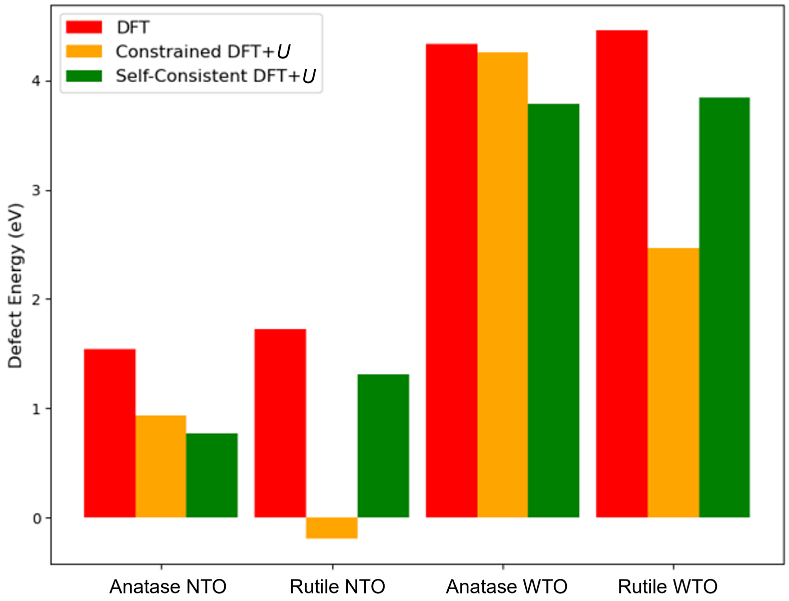

Figure S5: Defect energies for anatase and rutile NTO and WTO predicted using DFT, constrained DFT+U (U = 3 eV for anatase and 4 eV for rutile, $c_1 = 1$ and $c_2 = 0$) and self-consistent DFT+U (U = 3 eV for both anatase and rutile, $c_1 = 0.828$ and c_2 = -0.561).

The calculations in this manuscript were performed with FHI-aims versions 210618 and later, which were verified to yield consistent total energies and forces. After manuscript preparation, a minor error has been identified for the DFT+U implementation when used in combination with sparse matrix formats and hydrogenic basis functions. Specifically, an inconsistency has been identified between the occupation matrix and the corresponding Hamiltonian, due to an unintended double-counting of the Hubbard U correction on off-diagonal elements of the occupation matrix. To quantify the impact of this minor error, the self-consistent DFT+U simulations with the refined Hubbard projector were repeated with the corrected implementation, corresponding to the GitLab commit "a1ab632a0890b9a1c9373bbb1d75aa0f3faf4950", for both anatase and rutile NTO and WTO. The key differences in the localisation of polarons in anatase and rutile, as discussed in the density of states plots in Figure 3, are unchanged between the different code versions. The defect energies with the corrected implementation increase slightly for anatase NTO and WTO, by 0.08 eV and 0.17 eV, respectively; and decrease slightly for rutile NTO and WTO, by 0.23 eV and 0.20 eV, respectively. The quantitative differences noted for the different implementations do not affect the qualitative trends or conclusions as presented in the manuscript and are noted here for integrity purposes.

S2 Powder X-Ray Diffraction (XRD) Characterisation

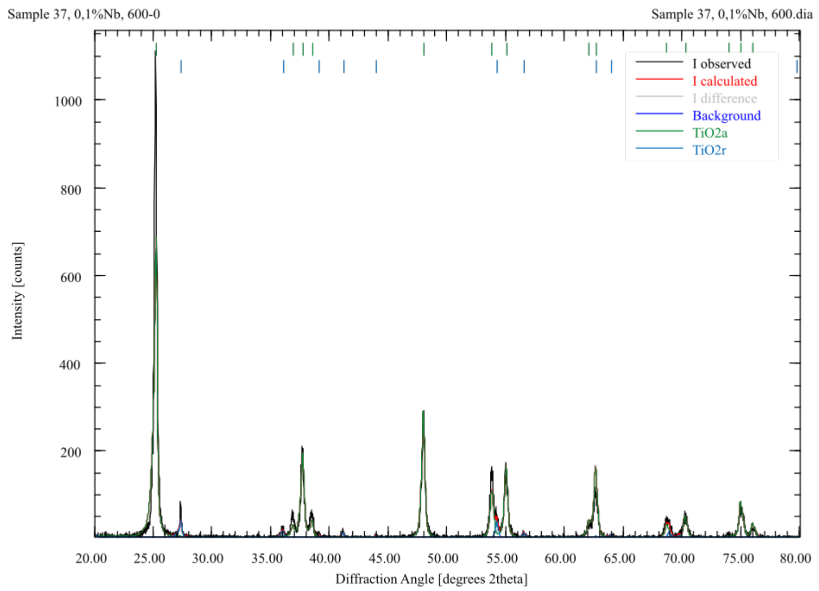

Figure S6: Refined powder XRD pattern of $0.1\,\%_{\mathrm{at.}}$ Nb doped TiO_2 (NTO-AR) indicating the presence of both anatase ($92\,\%$) and rutile ($8\,\%$) polymorphs.

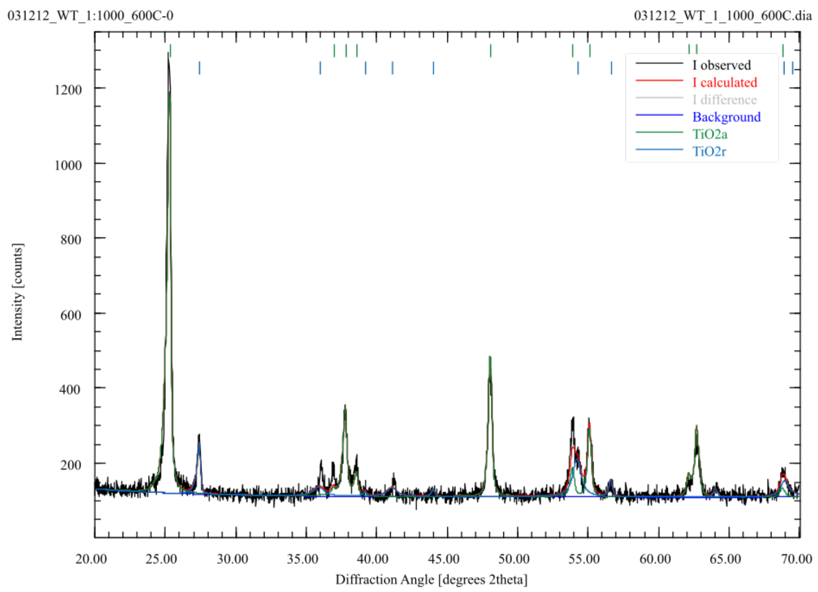

Figure S7: Refined powder XRD pattern of $0.1\,\%_{\text{at.}}$ W doped TiO_2 (WTO-AR) indicating the presence of both anatase ($72\,\%$) and rutile ($28\,\%$) polymorphs.

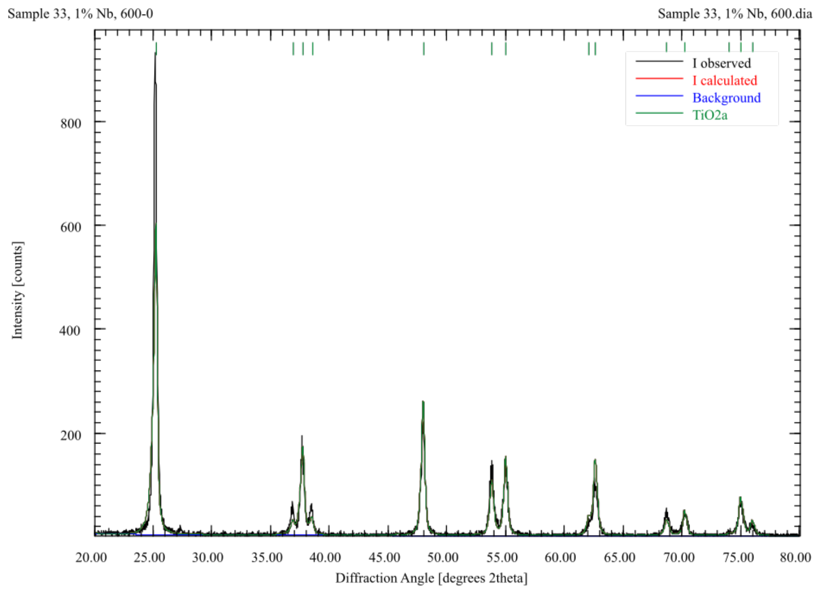

Figure S8: Refined powder XRD pattern of $1.0\,\%_{\text{at.}}$ Nb doped TiO_2 (NTO-A) indicating the presence of anatase only polymorph.

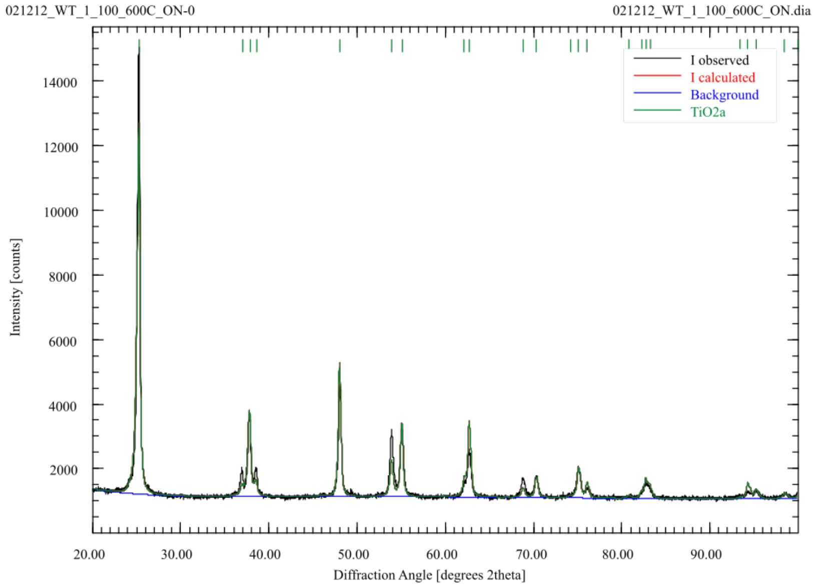

Figure S9: Refined powder XRD pattern of $1.0\,\%_{\mathrm{at.}}$ W doped TiO_2 (WTO-A) indicating the presence of anatase only polymorph.

Notes and references

1 T. Arlt, M. Bermejo, M. A. Blanco, L. Gerward, J. Z. Jiang, J. Staun Olsen and J. M. Recio, High-pressure polymorphs of anatase TiO_2, *Phys. Rev. B*, 2000, **61**, 14414–14419.

2 Y. Zhang, J. W. Furness, B. Xiao and J. Sun, Subtlety of TiO_2 phase stability: Reliability of the density functional theory predictions and persistence of the self-interaction error, *J. Chem. Phys.*, 2019, **150**, 014105.

3 M. Arrigoni and G. K. H. Madsen, A comparative first-principles investigation on the defect chemistry of TiO_2 anatase, *J. Chem. Phys.*, 2020, **152**, 044110.

4 L. Kavan, M. Grätzel, S. E. Gilbert, C. Klemenz and H. J. Scheel, Electrochemical and Photoelectrochemical Investigation of Single-Crystal Anatase, *JACS*, 1996, **118**, 6716–6723.

5 J. P. Allen and G. W. Watson, Occupation matrix control of d- and f-electron localisations using DFT+U, *Phys. Chem. Chem. Phys.*, 2014, **16**, 21016–21031.

6 M. Kick, K. Reuter and H. Oberhofer, Intricacies of DFT+U, Not Only in a Numeric Atom Centered Orbital Framework, *J. Chem. Theory Comput.*, 2019, **15**, 1705–1718.
